# Revision of the North African Hoverflies of the Genus *Xanthogramma* Schiner, 1861 (Diptera: Syrphidae), with Description of a New Species †

**DOI:** 10.3390/insects16080758

**Published:** 2025-07-23

**Authors:** Zorica Nedeljković, Ximo Mengual, Antonio Ricarte

**Affiliations:** 1 Research Institute CIBIO (Centro Iberoamericano de la Biodiversidad), Science Park, University of Alicante, Ctra. San Vicente del Raspeig s/n, 03690 San Vicente del Raspeig, Spain; ricarte24@gmail.com; 2 Museum Koenig Bonn, Leibniz Institute for the Analysis of Biodiversity Change, 53113 Bonn, Germany; x.mengual@leibniz-lib.de

**Keywords:** *Xanthogramma*, Palaearctic, Morocco, Tunisia, new species, new records, DNA barcoding, identification key

## Abstract

North Africa has a poorly and unevenly known hoverfly fauna. *Xanthogramma* Schiner, 1861 (Syrphinae, Syrphini) is represented in this territory by some scattered records of four species, *Xanthogramma dives* (Rondani, 1857), *Xanthogramma evanescens* Becker & Stein, 1913 (endemic to North Africa), *Xanthogramma marginale* (Loew, 1854), and *Xanthogramma pedissequum* (Harris, 1776). After examination of old *Xanthogramma* material collected in Tanger, Morocco, from the ‘Museo Nacional de Ciencias Naturales, Madrid, Spain (MNCN)’, specimens with distinctive morphology were spotted and found to be different from a syntype of *X. evanescens* collected in the same locality. Consequently, we revised all the available material of *Xanthogramma* from North Africa, characterised a new species, proposed a lectotype for *X. evanescens*, and provided an identification key to the North African species of this genus. The new species is also found in Tunisia and differs from *X. evanescens* in facial width, colour of the thoracic pleura, length of mesonotum hairs, wing pollinosity, and shape of the yellow maculae on tergum 2.

## Simple Summary

Hoverflies are a diverse family of insects (6000+ species) providing multiple ecosystem services and direct benefits to society, for example, in pollination and pest control. Although hoverflies are well studied in Europe and North America, these are poorly and unevenly known in North Africa. So, the aim of the present work is to gain knowledge on the diversity of the genus *Xanthogramma* in North Africa. We discovered and described one new species from Algeria and Tunisia. Our results are reinforced with DNA analysis to locate the studied species in a wider systematic framework within the genus *Xanthogramma*.

## 1. Introduction

The Palaearctic Region comprises territories in three different continents, Europe, Asia and Africa. In the latter of these three, the northern part shares biogeographical elements with the rest of the Palaearctic. Although the Palaearctic is the world region with the best-known insect diversity, within it there are less-studied areas such as North Africa [1]. North Africa is understood in this paper as the region from Egypt in the east to Morocco in the west and bordered in the south by the Sahara Desert.

The hoverflies or flower flies (Diptera, Syrphidae) provide a good number of ecosystem services, including the pollination of many plants and the regulation of populations of pest insects [2]. Despite their importance, hoverflies are still poorly and unevenly known in North Africa. In particular, the genus *Xanthogramma* Schiner, 1861 is known from this area just by some scattered records of four different species, namely *Xanthogramma dives* (Rondani, 1857), *Xanthogramma evanescens* Becker & Stein, 1913 (endemic to North Africa), *Xanthogramma marginale* (Loew, 1854), and *Xanthogramma pedissequum* (Harris, 1776) [3,4,5].

*Xanthogramma* flies are more or less slender, medium-sized hoverflies (8–14 mm, for the European species; [6]) with a typical black and yellow colour pattern. Both the nomenclature of this genus and its species’ taxonomy are complex [7]. There are 20 valid species worldwide [7,8,9,10], all Palaearctic except for one taxon found in the Nearctic Region, *Xanthogramma flavipes* (Loew, 1863) [11]. While adults of *Xanthogramma* can be spotted on flowers or hovering amongst vegetation, larvae are rarely observed and poorly understood, apparently predating root aphids in ant nests [12,13,14].

Upon the consultation of the old entomological collection of the ‘Museo Nacional de Ciencias Naturales, Madrid’, Moroccan specimens of a *Xanthogramma* species, supposedly similar to *X. evanescens* but with a unique combination of characters, was uncovered. Later on, a more recent specimen from Tunisia was also found in the collection of Museum Koenig Bonn, Germany. The finding of this species prompted us to revise the fauna of this hoverfly genus in North Africa and, as a result, to provide an updated overview of the species diversity of *Xanthogramma* in this poorly known Palaearctic area. In the present work, a new *Xanthogramma* species is described and figured, new records of this genus from North Africa are given together with DNA barcodes of *X. evanescens*, and an identification key for all North African species of *Xanthogramma* is provided.

## 2. Materials and Methods

All North African specimens of *Xanthogramma*, both published or not, found in various collections were examined in the framework of this study, including a syntype of *X. evanescens*. Material was deposited in the following collections (acronyms used in the examined material lists are also provided together with the name of the collection): Luomus, Finnish Museum of Natural History, Helsinki, Finland (MZH); Museo Nacional de Ciencias Naturales, Madrid, Spain (MNCN); Museum für Naturkunde, Berlin, Germany (ZMHB); Museum Koenig Bonn, LIB, Bonn, Germany (ZMFK); National Museum of Wales, Cardiff, United Kingdom (NMGW); entomological collection of the Université Larbi Tebessi, Tebessa, Algeria (ULT); personal collection of Martin J. Ebejer, Wales, United Kingdom (MEPC); personal collection of Sander Bot, The Netherlands (SBPC). Specimens were identified following [7,15]. In the examined material lists, a bar (‘/’) separates data from different labels.

**Figure 1:**
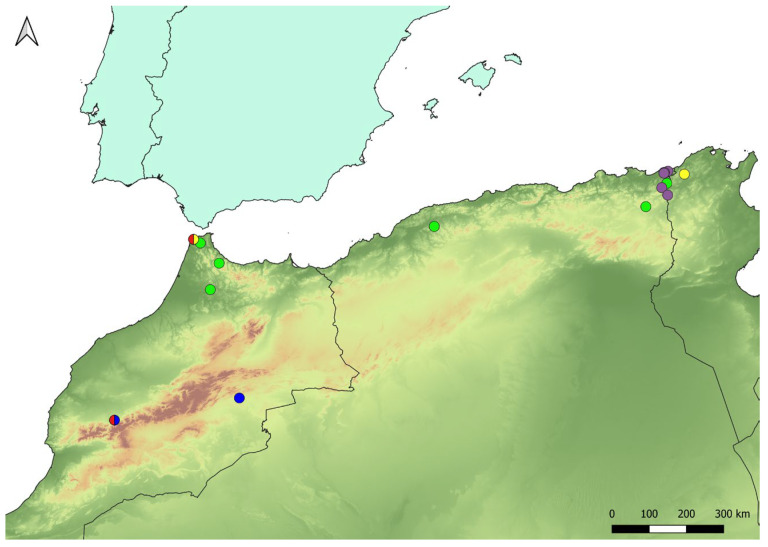
Map showing the North African distribution of *Xanthogramma* species. Yellow circle—*Xanthogramma africana* sp. n.; blue circle—*Xanthogramma dives*; red circle—*Xanthogramma evanescens*; Green circle—*Xanthogramma marginale*; violet circle—*Xanthogramma pedissequum*.

For the morphological study, colour characters always refer to dry specimens. Body length was measured from the tip of the frontal prominence (excluding antennae) to the tip of the abdomen. Wing length was measured as the length from the insertion point on the thorax to the tip of the wing. Antennal size was measured as a relation between the distance from the apex of the basoflagellomere and the most prominent point of the pedicel and the width of the basoflagellomere at the level of the arista base. Measurements were made using an eye-piece micrometre. Morphological terminology follows [16], except for the term “proepimeron” which follows [17], and the term ‘hair/s’ which is used instead of ‘pilis/pile’ of [16]. Photographs of specimens were taken with a Leica DFC 450 camera attached to a Leica M205 C binocular microscope. Male genitalia were dissected and prepared following [18]. A map representing the distribution of the examined specimens was produced with the software QGIS 3.28.10 [19] (Figure 1).

For the DNA barcoding [20,21], we followed the protocol by [22], as explained in [23]. One or two legs of selected specimens were used for DNA extraction and we kept the rest as DNA vouchers. All new sequences were submitted to GenBank via BOLD (www.boldsystems.org). In addition to our new sequences, we downloaded all the available public sequences of *Xanthogramma* from the Palaearctic Region in BOLD (accessed on 16 November 2024) and we included only the BOLD sequences with more than 500 nucleotides in our Neighbour-Joining (NJ) analysis. The DNA barcode of *Doros profuges* (Harris, 1780) (BOLD Process ID: GDIP1500-21) was constrained as the root for the NJ tree to facilitate the visualisation. Bootstrap support values (BS) were estimated from 1000 replicates directly from Geneious Prime ver. 2025.1.2 (Biomatters Ltd., Auckland, New Zealand). The NJ tree is provided in the Supplementary Materials, Figure S1. All sequences used in the present study are also publicly available in the BOLD dataset DS-XANTHOGR (“http://www.boldsystems.org/index.php/Public_SearchTerms?query=DS-XANTHOGR; accessed on 15 July 2025”) under the Digital Object Identifier dx.doi.org/10.5883/DS-XANTHOGR. 

## 3. Results

### 3.1. New Species Description

**Figure 2:**
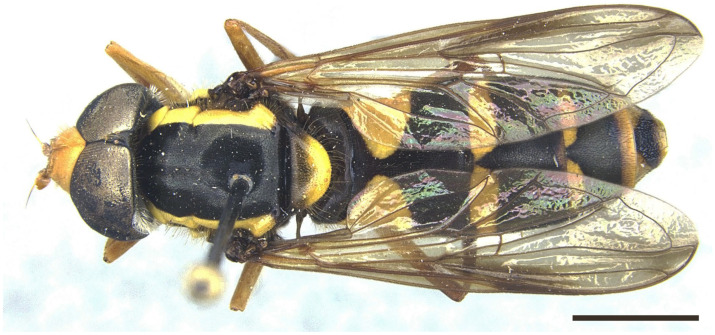
*Xanthogramma africana* sp. n., holotype, habitus dorsal view. Scale bar = 2 mm.

**Figure 3:**
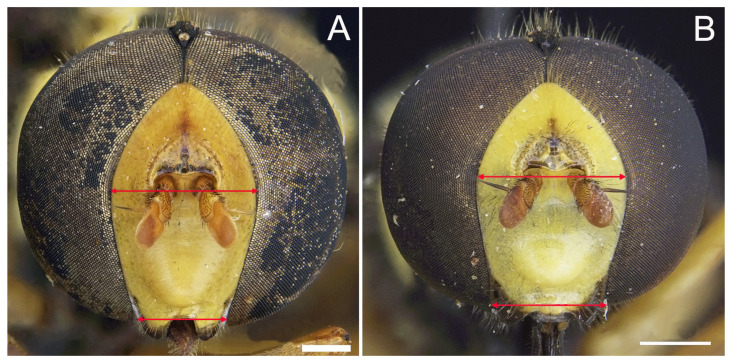
Head of *Xanthogramma* species, frontal view. (**A**): Head of *Xanthograma africana* sp. n. (**B**) Head of *Xanthogramma evanescens*. Arrows indicate face width. Scale bars = (**A**): 0.5 mm; (**B**): 0.75 mm.

**Figure 4:**
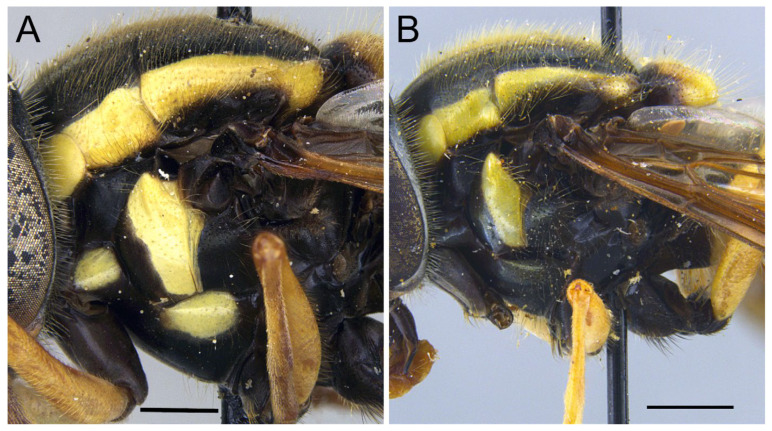
Pleura of *Xanthogramma* species, lateral view. (**A**): *Xanthogramma africana* sp. n. (**B**): *Xanthogramma evanescens*. Scale bars = (**A**): 0.75 mm; (**B**): 1 mm.

**Figure 5:**
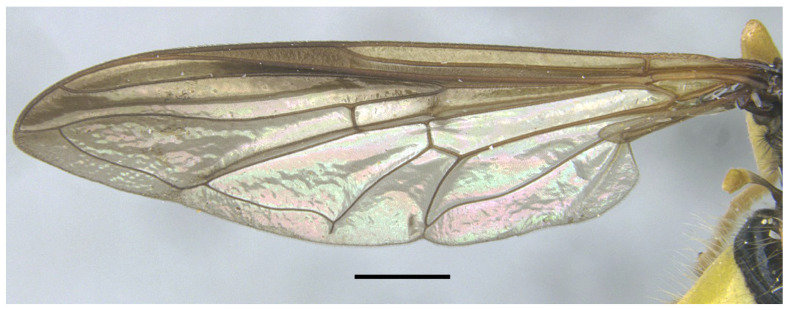
Wing of *Xanthogramma africana* sp. n. Scale bar = 1 mm.

**Figure 6:**
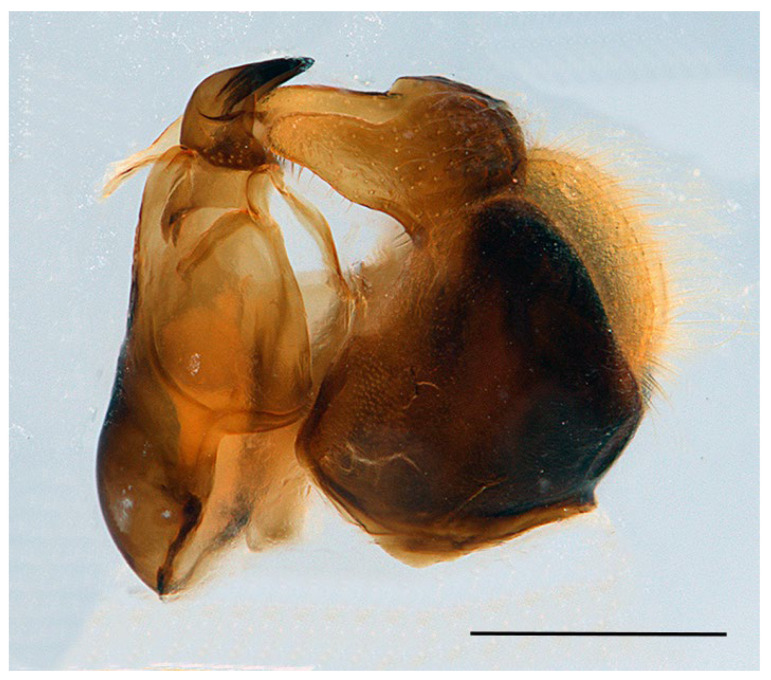
Male of *Xanthogramma africana* sp. n., genitalia, lateral view. Scale bar = 0.5 mm.

**Figure 7:**
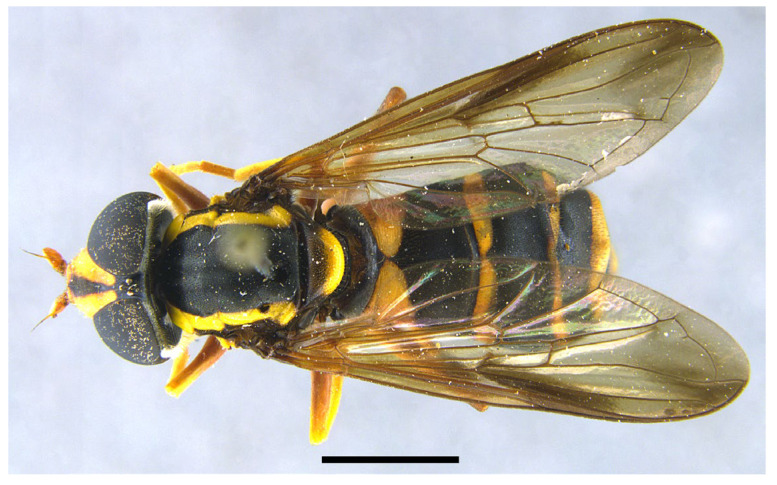
Female of *Xanthogramma africana* sp. n., habitus dorsal view. Scale bar =2 mm.

***Xanthogramma africana*** sp. n. (Figure 2, Figure 3A, Figure 4A, Figure 5, Figure 6 and Figure 7)

**urn:lsid:zoobank.org:pub:2A809DA8-0C5F-4E1B-A2D4-72BA681EC892**

Holotype

MOROCCO • ♂; Tanger, M. Escalera Leg./MNCN_Ent249779 (MNCN).

Paratypes

SPAIN• 5♂♂; Tanger; M. Escalera Leg./MNCN_Ent313116–313120 • 1♀; Tanger; M. Escalera Leg./MNCN_Ent249778 (MNCN) • 1♂; TUNISIA; Jendouba Governorate; 10 km E of Aïn Draham; 36°46′ N 08°55′ E; 13 June 2000; M. Hauser Leg./ZFMK-DIP-00082481 (ZFMK).

*Diagnosis*. Eye bare; proepimeron with a yellow macula; posterior anepisternum, posteriorly, with a yellow macula; katepisternum, dorsally, with a yellow macula; pro- and mesolegs completely yellow (tibiae and tarsi dark yellow); hind tibia with a black median ring; wing microtrichose except anterior part of cell bm, which is bare; cell r_1_, medially, with a brown macula; cell r_2+3_, apically, with brown pigmentation; wing with two separated dark brown patches connected with light brown pigmentation; tergum 2 with two triangular fasciae occupying more than half of the tergum width, fasciae with oval inner margin and reaching the lateral margins of the tergum.

*Description*. MALE (holotype) (Figure 2). Body length = 13.17 mm; wing length = 9.76 mm. Head (Figure 3A). Eye bare (only some scattered short yellow hairs can occur on its dorsal part); vertical triangle black with black pollinosity and yellow hairs; ocellar triangle isosceles; occiput grey pollinose along eye margin, with long yellow hairs; frontal triangle yellow with long yellow hairs dorsally and some short black hairs ventrally (near lunules); lunule transparent yellow; antenna yellow, scape and pedicel with short black hairs; basoflagellomere oval (about 1.2× longer than broad); arista yellow basally and dark yellowish-orange apically, with short sparse hairs (hairs shorter that the arista diameter); face yellow with yellow hairs; gena black laterally, yellow (with yellow hairs) medially; eye contiguity about 2.6× shorter than height of frontal triangle. Thorax. Scutum black with two white pollinose vittae extending for the anterior two thirds of the scutum length; scutum with long erect yellow hairs intermixed with short adpressed yellow hairs; notopleuron yellow, narrowly black near the wing base; proepimeron with a yellow macula; posterior anepisternum with a yellow macula on its posterior part; katepisternum, dorsally, with a yellow macula (Figure 4A); scutellum transparent black on its anterior margin, matt black at the lateral corners and yellow on the posterior margin, with long yellow hairs; coxae and trochanters black; pro- and mesofemora yellow, pro- and mesotibiae dark yellow; pro- and mesotarsi dark yellow; metatibiae with a black median ring; wing microtrichose, except for the anterior part of cell bm, which is bare, and the posterior part of cell r having sparse microtrichia; pterostigma dark brown, cell r_1_, medially, with a brown macula; cell r_2+3_ with brown pigmentation apically; wing with two dark brown maculae interconnected by light brown microtrichia (Figure 5). Abdomen (Figure 2). Shiny black with short black hairs, except for tergum 1, anterior part of tergum 2, and yellow maculae on all terga that bear yellow hairs; tergum 2 with two triangular maculae occupying more than half of the tergum width, maculae with rounded tips and reaching the lateral margins of the tergum; tergum 3 with two yellow fasciae reaching its lateral margins, very close to each other in the medial part of the tergum and occupying more than the anterior fourth of the tergum width; tergum 4 with two separate yellow fasciae not reaching the lateral margin; posterior margin of tergum 4 with yellow fasciae not reaching the lateral margins; tergum 5 with two yellow maculae very close each other in the medial part of the tergum, posterior margin of tergum 5 with a yellow fascia reaching the lateral margins; sternum 1 black in the anterior two thirds, yellow in the posterior third, covered with long yellow hairs; sternum 2 yellow in the anterior two thirds, black in the posterior third, and yellow on the posterior margin, covered with long yellow hairs and, in the posterior part of the black fascia, long black hairs; sternum 3 black, only yellow in the anterior fourth, yellow fascia covered with yellow hairs and black part of the sternum with black adpressed hairs; sternum 4 black with two yellow maculae on its anterior part; sternum 5 completely black with black adpressed hairs. Genitalia (Figure 6). Surstylus beak-shaped. Hypandrium 2.5 × longer than wide.

FEMALE (Figure 7). Body length = 10.17 mm. Similar to male except for the following characters: occiput with mainly yellow hairs, but some black hairs on the area posterior to the ocellar triangle; black vitta extending from ocellar triangle to the lunule, the black vitta bearing yellow hairs; ocellar triangle with yellow hairs.

*Etymology*. The specific epithet ‘africana’ means ‘from Africa’ in Latin and refers to the continent of the type locality. To be treated as adjective.

*Range*. Morocco and Tunisia.

*Biology*. Adults fly from May to June. Larva unknown but suspected to be aphidophagous.

**Figure 8:**
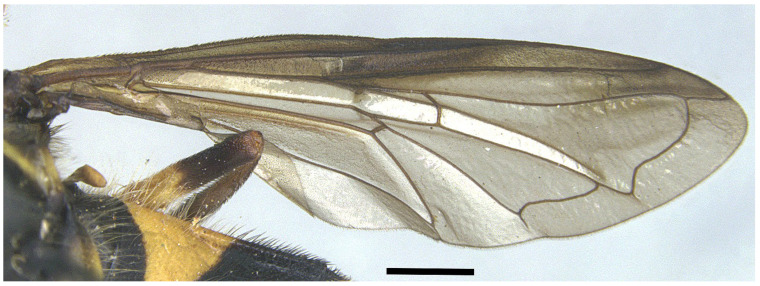
Wing of *Xanthogramma evanescens*. Scale bar = 1 mm.

**Figure 9:**
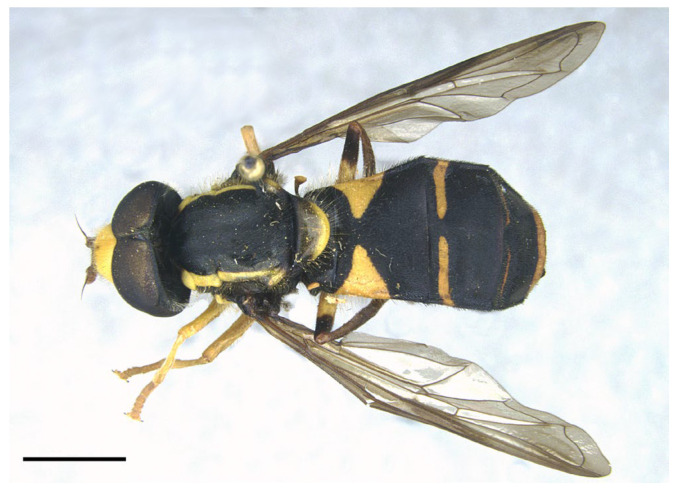
*Xanthogramma evanescens*, lectotype, habitus dorsal. Scale bar = 2 mm.

*Taxonomic notes*. *Xanthogramma africana* sp. n. is similar to *X. evanescens* and can be distinguished from it by the presence of a yellow macula on the proepimeron and katepisternum (Figure 4A), which are completely black in *X. evanescens* (Figure 4B). The mesonotum pilosity in *X. africana* sp. n. consists of both long erect and semi-recumbent shorter hairs intermixed, whilst there are only long erect hairs in *X. evanescens*. The facial width also differs in these two species, being conspicuously narrower in *X. africana* sp. n. (Figure 3A) than in *X. evanescens* (Figure 3B). The anterior part of cell bm is bare in *X. africana* sp. n., but sparsely microtrichose in *X. evanescens*. In *X. africana* sp. n., the wing has two brown maculae that can be either conspicuously separated or interconnected by a faint line of light brown pigmentation (Figure 5), but in *X. evanescens* the two maculae are conspicuously interconnected by a dark brown line (Figure 8). Regarding the abdomen, *X. africana* sp. n. has the triangular yellow maculae of tergum 2 with round inner ends (Figure 2), whilst they are more acute in *X. evanescens* (Figure 9); the yellow maculae of the tergum 2 cover about half of the length of the lateral margin of the tergum in *X. africana* sp. n., but about a third of the lateral margin in *X. evanescens*. Yellow fasciae on tergum 3 occupy a sixth of tergite width in *X. evanescens*, but about a fourth of tergite width in *X. africana* sp. n. Tergum 4 has very thin yellow fasciae in *X. evanescens*, occupying a tenth of tergum width, whilst in *X. africana* sp. n., the yellow fasciae occupy a fourth of tergum width.

### 3.2. Other North African Species of Xanthogramma

*Xanthogramma dives* (Rondani, 1857)

Published records (not revised). Morocco [5,24].

*Examined material. New.* MOROCCO • 1*♂*, 1*♀*; Errachidia; Eastern High Atlas; 1570 m; 29 km N of Rich; 32°26.679′ N, 4°29.275′ W; 4 May 2012, M.J. Ebejer Leg./DNA voucher specimen, ZFMK, Lab code D541; Bonn, Germany (*♂*); DNA voucher specimen, ZFMK, Lab code D542, Bonn, Germany (*♀*) (MEPC). • 1*♂*; MA Marrakech-Safi, near Asni; 1068 m; gps 31.2889, −7.9622; 14 March 2023; S. Bot Leg./Bonn Bot 358/*Xanthogramma evanescens* Becker, 1913; S. Bot Det.; 2023 (SBPC).

*Remarks*. We tried to obtain DNA from the two specimens from Errachidia, but the amplification failed.

*Xanthogramma evanescens* Becker, 1913 (Figure 8 and Figure 9).

Published records (not revised). Morocco [5,8,25].

*Examined material*. LECTOTYPE (designated here): 1*♂*; “Typus” [red label]/1897 Tanger [white label]/*Xanthogramma evanescens* B (hand written) Becker Det.; [white label]/1897 [red triangular label]/Zool.Mus. Berlin [white label]/Sammlung Dr. Th Becker [white label]/LECTOTYPUS *Xanthogramma evanescens* Becker design. C. Kassebeer 1992 [yellow label]. *New*. MOROCCO, 4*♂♂*; MA Marrakech-Safi, near Asni; 1068 m; gps 31.2889, *−*7.9622; 14 March 2023; S. Bot Leg./*Xanthogramma evanescens* Becker, 1913, S. Bot Det.; 2023/Bonn Bot 334 (ZFMK-DIP-00100583)/Bonn Bot 356 (ZFMK-DIP-00100523)/Bonn Bot 357 (ZFMK-DIP-00100524)/Bonn Bot 358 (ZFMK-DIP-00100525) (SBPC).

*Remarks*. A lectotype is designated here for *X. evanescens* in order to stabilise this species concept, since it could be confused with other similar species (*Xanthogramma dives*). The specimen here designated as lectotype was already labelled as ‘lectotype’ by Christian Kassebeer, but, to the best of our knowledge, it was never published. We confirm this specimen belongs to the type series of *X. evanescens* designated by [15].

We obtained four DNA barcodes (GenBank accession numbers: EUROP138-24, EUROP139-24, EUROP140-24, EUROP142-24) for the specimens collected in Marrakech-Safi (Morocco).

*Xanthogramma pedissequum* (Harris, 1778)

*Published records (not revised)*. Morocco [5,25,26].

**Figure 10:**
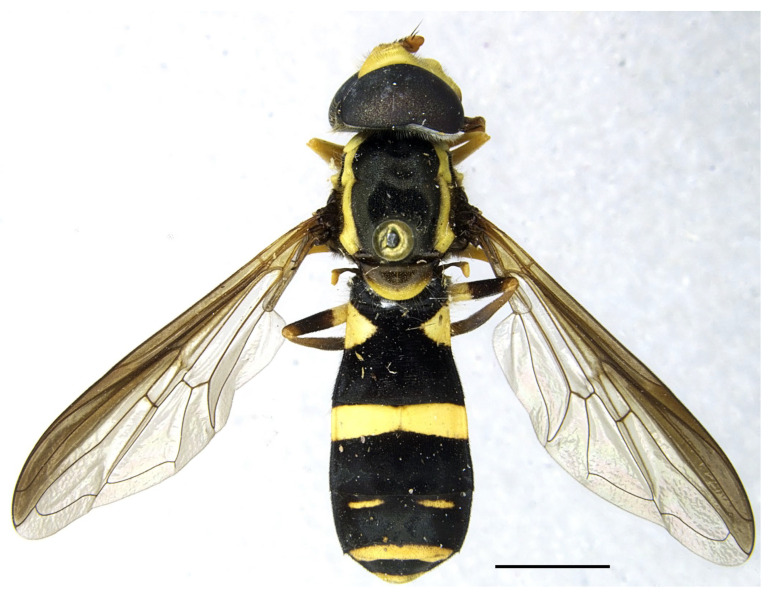
*Xanthogramma marginale*, habitus dorsal view. Scale bar = 2 mm.

*Xanthogramma marginale* (Loew, 1854) (Figure 10).

*Published records (not revised)*. Morocco [5,15,27,28,29,30,31].

*Published records (revised)*. Algeria [4].

*Examined material. New.* MOROCCO: 1*♂*; Tanger; M. Escalera Leg./MNCN_Ent 313122. • 1*♂*; Tanger; M. Escalera Leg./*Xanthogramma marginale* Loew, Gil Collado det./MNCN_Ent 313121 (MNCN). • 1*♂*, Atlas mal., Arround, 9-12.6.26, Lindberg/Gj. 2078 Loan/Gj. 2078 Loan http://tun.fi/HRA.27254.

### 3.3. Key to the North African Species of Xanthogramma

1. Tergum 2 elongated, almost 2× longer than the basal tergite’s width (Figure 10) … …………………………………………………………….*X. marginale* [Algeria, Morocco].
  - Tergum 2 shorter (up to 1.5× longer than the basal tergite’s width) (Figure 2)… .… 2.
2. Tergum 4 with very narrow yellow fasciae, occupying about a fifth of the tergite width ... ……………………………………………………………………………………..3.
  - Tergum 4 with wider yellow fasciae, occupying about a fourth of the tergite width ……………………………………………………………………………………………4.
3. Pleura: only anterior anepisternum with a yellow macula (Figure 4B); face at the level of antennal insertions 1.3× wider than face at the level of mouth edge (Figure 3B); triangular yellow macula of tergite 2 with narrower and more pointed inner end (Figure 8) ... ……………………………………………………………*X. evanescens* [Morocco].
  - Pleura: proepimeron, anterior anepisternum, and katepisternum with yellow maculae (Figure 4A); face at the level of the antennal insertions 1.7× wider than the face at the level of mouth edge (Figure 3A); triangular yellow macula of tergum 2 with broader and blunter inner end (Figure 2)………….*X. africana* sp. n. [Morocco, Tunisia].
4. Thoracic pleura with 1–2 yellow maculae laterally ............... *X. pedissequum* [Morocco].
  - Thoracic pleura with more than 2 yellow maculae laterally .............. *X. dives* [Morocco].

### 3.4. Molecular Study

A total of 100 DNA barcodes belonging to 11 *Xanthogramma* species were included in the molecular analyses (see Figure S1 of Supplementary Materials). The newly obtained COI sequences of *X. evanescens* clustered together with other sequences of the same species (BS = 93.1%), but as shown previously [7], the COI sequences of the different putative species were not resolved in different clusters with high support.

## 4. Discussion

The results based on the morphological analysis of *Xanthogramma* specimens revealed the presence of five well-differentiated species in North Africa, including *X. africana* sp. n. The new species can be distinguished from the similar *X. evanescens*, for instance, by the presence of a yellow macula on the proepimeron and katepisternum (Figure 4A), which are completely black in *X. evanescens* (Figure 4B), and the mesonotum hairs, which are both long erect and shorter semi-recumbent intermixed in *X. africana* sp. n., but only long in *X. evanescens*. Some morphological characters, such as the colour of the thoracic pleura, have proven useful, once more, in separating *Xanthogramma* species; this character is also useful to separate *X. pedissequum/X. dives, X. pilosum/X. laetum*, and *X. aeginae/X. citrofasciatum* [7]. Although we tried to sequence individuals of our new species, *X. africana* sp. n., in order to compare the DNA barcodes with *X. evanescens*, we were only able to obtain molecular data for *X. evanescens*. In the phylogenetic three produced, the poor utility of COI alone to separate *Xanthogramma* species is evidenced (Figure S1).

The new species apparently coexist with *X. evanescens*, as both have been recorded in Tanger, in Morocco [15]. In addition, *X. evanescens* has also been reported from Tunisia [5], but we have not been able to access this material to confirm its identity. *Xanthogramma marginale* occurs in Morocco and Algeria [4,5,26], *X. pedissequum* also in Morocco and Algeria [3,27], and *X. dives* in the High Atlas of Morocco [24].

The taxonomic complexity of *Xanthogramma* and the scattered records of hoverflies of this genus from North Africa, together with the finding of *X. africana* sp. n., suggest that the genus is under-sampled in this part of the Palaearctic Region and that new congeneric species might be awaiting discovery. In addition, the recent finding and description of overlooked species with unique morphology in old collections [32,33] indicate the need to promote the examination and study of entomological collections as a source of important taxonomic and faunistic data, as well as for their use in phylogenomics (museomics and collectomics [34]).

## 5. Conclusions

Morphological and molecular evidence was combined to update the taxonomy and systematics of North African *Xanthogramma* species. According to the main aim of this work, the conclusions are as follows:

(1) A total of five *Xanthogramma* species are present in North Africa.
(2) One new North African species, *X. africana* sp. n., is described and illustrated.

This is the first work devoted to update the taxonomy, systematics, and distribution of the genus *Xanthogramma* in the African continent.

## Data Availability

All sequences generated in this work are available in the publicly accessible repository of GenBank (“https://www.ncbi.nlm.nih.gov/genbank/ (accessed on 15 July 2025)”) and BOLD (“http://www.boldsystems.org/index.php/Public_SearchTerms?query=DS-XANTHOGR (accessed on 15 July 2025)”).

## Supplementary Materials

The following supporting information can be downloaded at https://www.mdpi.com/article/10.3390/insects16080758/s1. Figure S1: Neighbour-Joining tree using Jukes–Cantor model based on COI sequences of *Xanthogramma*, with *Doros profuges* (Harris, 1780) constrained as the outgroup. Bootstrap support values (>85%) are indicated at the nodes. The name for each specimen has the name of the species | sample ID | country of origin | GenBank accession number or BOLD Process ID.

## References

[1] Konstantinov A.S., Korotyaev B.A., Volkovitsh M.G., Foottit R., Adler P. (2009). Insect biodiversity in the Palearctic Region. Insect Biodiversity: Science and Society.

[2] Rotheray G.E., Gilbert F. (2011). The Natural History of Hoverflies.

[3] Djellab S., van Eck A., Samraoui B. (2013). A survey of the hoverflies of northeastern Algeria (*Diptera: Syrphidae*). Egypt. J. Biol..

[4] Mebarkia N., Neffar S., Djellab S., Ricarte A., Chenchouni H. (2021). New records, distribution and phenology of hoverflies (*Diptera: Syrphidae*) in semi-arid habitats in northeastern Algeria. Orient. Insects.

[5] Sahib S., Driauach O., Belqat B. (2020). New data on the hoverflies of Morocco (*Diptera, Syrphidae*) with faunistic and bibliographical inventories. Zookeys.

[6] Speight M.C.D. (2020). Species accounts of European Syrphidae, 2020. Syrph the Net, the Database of European Syrphidae (Diptera).

[7] Nedeljković Z., Ricarte A., Šašić Zorić L., Đan M., Obreht Vidaković D., Vujić A. (2018). The genus *Xanthogramma* Schiner, 1861 (*Diptera: Syrphidae*) in southeastern Europe, with descriptions of two new species. Can. Entomol..

[8] Peck L.V., Soos A., Papp L. (1988). Syrphidae. Catalogue of Palaearctic Diptera.

[9] Huo K.K., Ren G.D., Zheng Z.M. (2007). Fauna of Syrphidae from Mt. Qinling-Bashan in China (Insecta: Diptera).

[10] Barkalov A.V., Mutin V.A. (2008). Checklist of the hover-flies (*Diptera, Syrphidae*) of Russia. Euroasian Entomol. J..

[11] Evenhuis N.L., Pape T. Systema Dipterorum, Version 2.10. http://diptera.org/.

[12] Hölldobler K. (1929). Uber die Entwicklung der Schwierfleige *Xanthogramma citrofasciatum* im Neste von *Lasius alienus* und *niger*. Zool. Anzeig..

[13] Dixon T.J. (1960). Key to and descriptions of the third instar larvae of some species of Syrphidae (*Dipt.*) occurring in Britain. Trans. R. ent. Soc. Lond..

[14] Rotheray G.E., Barr B., Hewitt S.M. (1996). The myrmecophilous larvae of *Chrysotoxum arcuatum*, *Pipizella varipes* and *Xanthogramma pedisequum* from Europe and *Platycheirus milleri* from New Zealand (*Dip.: Syrphidae*). Entomol. Rec. J. Var..

[15] Becker T., Stein P., Adelung N. (1913). Dipteres aus Marokoo. Annuaire du Musée Zoologique de l’ Academie Imperiale des Sciences de St.-Pétersbourg.

[16] Thompson F.C. (1999). A key to the genera of flower flies of the Neotropical Region including the descriptions of genera and species and glossary of taxonomic terms. Contrib. Entomol. Int..

[17] Speight M.C., Sarthou J.P. (2017). StN keys for the identification of the European species of various genera of Syrphidae, 2017. Syrph the Net, the Database of European Syrphidae (Diptera).

[18] Ricarte A., Nedeljković Z., Rotheray G.E., Lyszkowski R., Hancock E., Watt K., Hewitt S., Horsfield D., Wilkinson G. (2012). Syrphidae (*Diptera*) from the Greek island of Lesvos, with description of two new species. Zootaxa.

[19] QGIS Geographic Information System.

[20] Hebert P.D.N., Cywinska A., Ball S.L., de Waard J.R. (2003). Biological identifications through DNA barcodes. Proc. R. Soc. Lond. B.

[21] Hebert P.D.N., Ratnasingham S., De Waard J.R. (2003). Barcoding animal life: Cytochrome c oxidase subunit 1 divergences among closely related species. Proc. R. Soc. Lond. B.

[22] Rozo-Lopez P., Mengual X. (2015). Mosquito species (*Diptera, Culicidae*) in three ecosystems from the Colombian Andes: Identification through DNA barcoding and adult morphology. ZooKeys.

[23] Bot S., Mengual X., van Steenis J., Skevington J.H. (2022). A new species of the genus *Milesia* Latreille (*Diptera: Syrphidae*) from Crete. Eur. J. Taxon..

[24] Ebejer M.J., Kettani K., Gatt P. (2019). First records of families and species of Diptera (*Insecta*) from Morocco. Boletín de La S.E.A..

[25] El-Hawagry M.S., Gilbert F. (2019). Catalogue of the Syrphidae of Egypt (*Diptera*). Zootaxa.

[26] Dirickx H.G. (1994). Atlas des Diptères Syrphides de la Région Méditerranéenne 75.

[27] Gil Collado J. (1929). Sírfidos de Marruecos del Museo de Madrid. (*Dipt. Syrph*.). Mem. de La Real. Soc. Española De Hist. Nat..

[28] Kanervo E. (1930). Inventa entomologica itineris Hispanici et Maroccani, quod a. 1926 fecerunt Harald et Hakan Lindberg. Comment. Biol..

[29] Claussen C., Hauser M. (1990). Neue Syrphidenvorkommen aus Marokko und Tunesien (*Diptera, Syrphidae*). Entomofauna.

[30] Séguy E. (1961). Dipteres Syrphides de l’Europe Occidentale, Série A.

[31] Claussen C. (1989). Syrphiden aus Marokko (*Diptera, Syrphidae*). Entomofauna.

[32] Aguado Aranda P., Ricarte A., Nedeljković Z., Marcos-García M.A. (2022). An overlooked case for a century: Taxonomy and systematics of a new Iberian species of *Eumerus* Meigen, 1822 (*Diptera, Syrphidae*). Eur. J. Taxon..

[33] Ricarte A., Nedeljković Z., Aguado Aranda P., Marcos García M.A. (2022). Assessing the Diversity and Systematics of Brachyopini Hoverflies (*Diptera: Syrphidae*) in the Iberian Peninsula, Including the Descriptions of Two New Species. Insects.

[34] Kapun M., Schwentner M., Haring E., Akkari N., Kroh A., Kruckenhauser L., Palandacic A., Vohland K. (2025). Museomics, the Extended Specimen and Collectomics—How to frame and name the diversity of information linked to specimens in natural history collections?. arXiv.

