# Supplementary material for: Revision of the North African Hoverflies of the Genus Xanthogramma Schiner, 1861 (Diptera: Syrphidae), with Description of a New Species
Source: Insects. 2025 Jul 23;16(8):758. doi: 10.3390/insects16080758 (PMC12386446; doi:10.3390/insects16080758)
Supplement: Supplementary file 1 [file insects-16-00758-s001.zip › insects-3754869-supplementary.pdf]

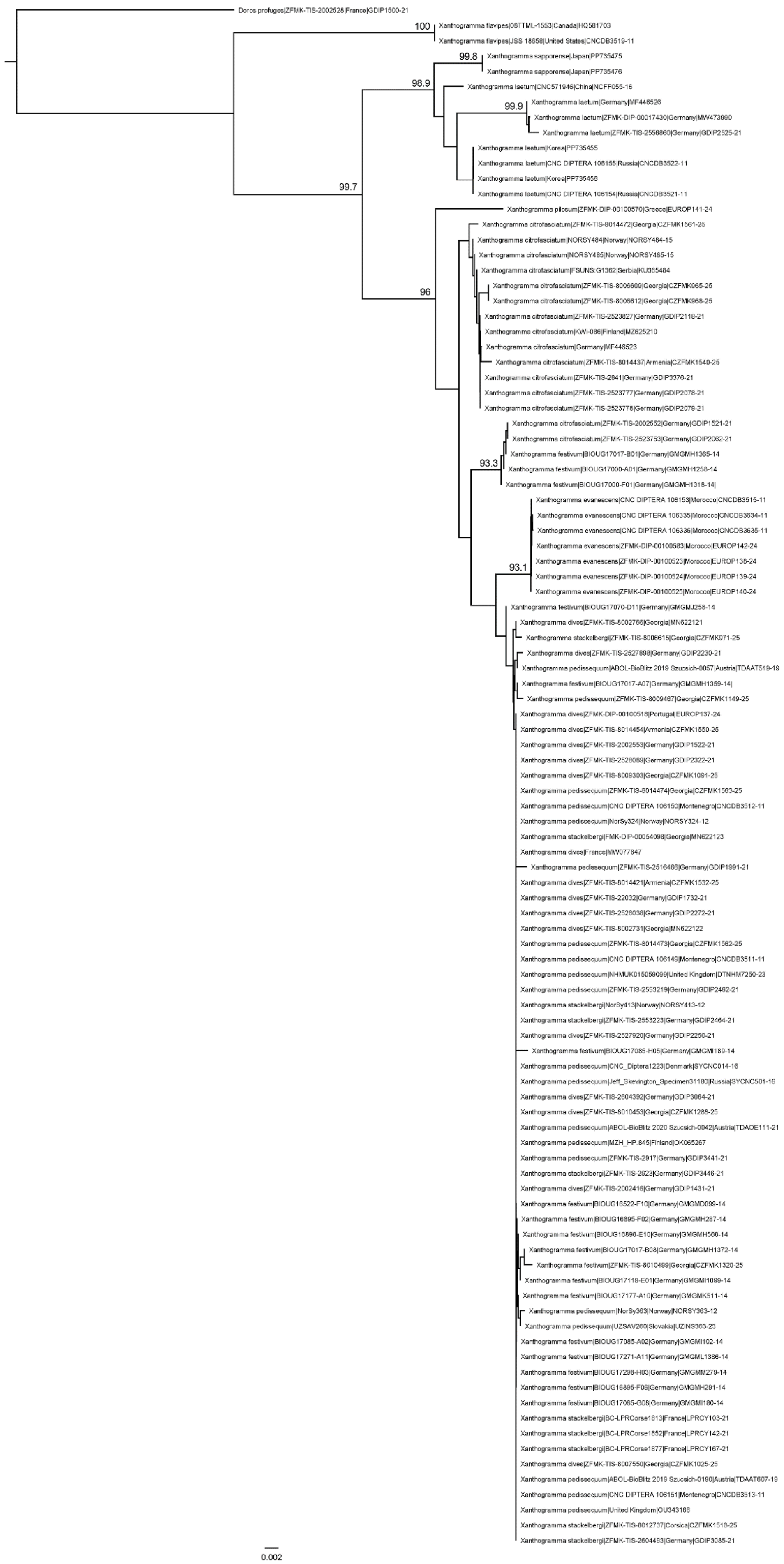

Figure S1. Neighbor-Joining tree using Jukes-Cantor model based on COI sequences of *Xanthogramma*, with *Doros profuges* (Harris, 1780) constrained as the outgroup. Bootstrap support values (>85%) are indicated at the nodes. The name for each specimen has: the name of the species | sample ID | country of origin | GenBank accession number or BOLD Process ID.
